# Supervised Machine Learning-Based Prediction of Hydrogen Storage Classes Utilizing Dibenzyltoluene as an Organic Carrier

**DOI:** 10.3390/molecules29061280

**Published:** 2024-03-13

**Authors:** Ahsan Ali, Muhammad Adnan Khan, Hoimyung Choi

**Affiliations:** 1 Department of Mechanical Engineering, Gachon University, Seongnam 13120, Republic of Korea; ahsanali@gachon.ac.kr; 2 School of Computing, Skyline University College, University City Sharjah, Sharjah 1797, United Arab Emirates; muhammad.adnan@skylineuniversity.ac.ae; 3 Riphah School of Computing & Innovation, Faculty of Computing, Riphah International University Lahore Campus, Lahore 54000, Pakistan; 4 Department of Software, Faculty of Artificial Intelligence and Software, Gachon University, Seongnam 13120, Republic of Korea

**Keywords:** 5-Fold Cross Validation, Holdout Validation, HSP-SVM, Resubstitution Validation, Support Vector Machine

## Abstract

Dibenzyltoluene (H0-DBT), a Liquid Organic Hydrogen Carrier (LOHC), presents an attractive solution for hydrogen storage due to its enhanced safety and ability to store hydrogen in a concentrated liquid form. The utilization of machine learning proves essential for accurately predicting hydrogen storage classes in H0-DBT across diverse experimental conditions. This study focuses on the classification of hydrogen storage data into three classes, low-class, medium-class and high-class, based on the hydrogen storage capacity values. We introduce Hydrogen Storage Prediction with the Support Vector Machine (HSP-SVM) model to predict the hydrogen storage classes accurately. The performance of the proposed HSP-SVM model was investigated using various techniques, which included 5-Fold Cross Validation (5-FCV), Resubstitution Validation (RV), and Holdout Validation (HV). The accuracy of the HV approach for the low, medium, and high class was 98.5%, 97%, and 98.5%, respectively. The overall accuracy of HV approach reached 97% with a miss clarification rate of 3%, whereas 5-FCV and RV possessed an overall accuracy of 93.9% with a miss clarification rate of 6.1%. The results reveal that the HV approach is optimal for predicting the hydrogen storage classes accurately.

## 1. Introduction

The renewable energy sources are receiving great attention in the modern world due to gradual increments in the energy demand as the global population is increasing. The global population is expected to reach a figure of 10 billion by 2050 [1]. Energy needs are increasing in the world, and countries are turning to renewable energy resources as well as fossil fuels to meet their needs. In the coming years, the utilization of energy will increase exponentially. There is a limit to the life of fossil fuels, so finding new energy sources is important. Global warming poses a significant challenge due to the adverse impacts associated with the utilization of fossil fuels, including oil, coal, and natural gas [2]. The utilization of fossil fuels for power generation is progressively diminishing in developed nations. It is quite difficult to replace fossil fuels immediately because fossil fuels meet 80% of our everyday energy demands [3]. According to a report by the World Health Organization (WHO), fossil fuels usage contributes to climate change, which has negative impacts on human health [4,5]. This has made it even more essential to reduce fossil fuel use using renewable energy sources. Renewable energy has a lower environmental effect than traditional energy conversion techniques; it is considered a clean energy source with nearly no carbon emissions [6]. Every human activity has the potential to affect the environment; nonetheless, when considering environmental implications, renewable approaches should be favored above other methods.

Hydrogen has emerged as an efficient form of energy storage that produces zero carbon emissions, making it an environmentally friendly option. Moreover, its energy content (141.7 MJ/Kg) is higher than that of fossil fuels (45.8 MJ/Kg). Hydrogen energy possesses almost seven times higher gravimetric density than fossil fuels [7]. These characteristics of hydrogen energy make it a favorable energy source for the future. However, hydrogen has a low volumetric density, which makes it quite difficult to store. Commercially used hydrogen storage techniques, such as cryogenic storage and pressurized gas storage, have the disadvantages of requiring high amounts of energy, experiencing boil-off losses, and being difficult to transport [8,9,10].

The Liquid Organic Hydrogen Carriers (LOHCs) system is seen as a suitable approach for storing hydrogen into aromatic compounds. This system elevates the boil-off losses and transport issues. Several LOHC systems have been investigated to find an efficient system. Some of the efficient LOHC systems are the carbazole [11,12,13,14,15,16,17], indole [18,19,20,21,22], and acridine [23] derivatives. The gravimetric hydrogen density of N-ethylcarbazole (NEC) is 5.8 wt.%, which makes it an efficient LOHC system. However, it carries a major drawback of solidifying at room temperature. Brückner et al. [24] introduced a Dibenzyltoluene (H0-DBT) and perhydro-Dibenzyltoluene (H18-DBT) pair in 2014. H0-DBT eliminates the solidification concern as it is present in liquid form. In recent years, researchers have focused on H0-DBT due to its high gravimetric storage density of 6.2 wt.% [25,26,27,28,29,30]. It also possesses reversibility characteristics, and the hydrogen is produced during a dehydrogenation reaction. These characteristics reveal that H0-DBT is an efficient candidate for storing hydrogen in a wide range of applications. 

The several studies focusing on the hydrogenation of H0-DBT have been reported to highlight the attained hydrogen storage capacity under various experimental conditions [24,28,31,32]. The hydrogenation reaction is influenced by several key parameters such as the reaction temperature, initial pressure, and ratio of catalyst to H0-DBT. The hydrogen storage capacity of H0-DBT is varied when the reaction conditions, such as the reaction temperature and initial pressure, are different. Categorizing hydrogen storage in H0-DBT based on storage capacity can help identify the various classes of stored hydrogen. Machine learning algorithms (MLAs) have been employed recently to analyze the available data and make more accurate predictions for hydro-gen storage. This approach can assist to identify the optimal reaction parameters for hydrogenation of H0-DBT and other LOHCs in a short time and minimize the efforts of researchers. 

Several materials, such as electrocatalysts [33,34], perovskite solids [35,36,37,38], thermoelectric [39,40,41,42], interphase precipitation in micro-alloyed steels [43], carbon-capture materials [44,45], light-emitting transistors [46], and oxides and inorganic materials [47,48,49,50], have been considered in recent times to apply the MLAs. The various machine learning models have been applied for predicting the adsorption behavior of H_2_, CH_4_, C_3_H_8_, and CO_2_ in H_2_-selective nanocomposite membranes. The results elucidated that the Committee Machine Intelligent System (CMIS) exhibited the highest accuracy in comparison to another model with R2 = 0.9997 [51]. Rezakazemi et al. employed the genetic algorithm (GA) and particle swarm optimization (PSO) to enhance the performance of adaptive neuro-fuzzy inference system (ANFIS), which was used to study the performance of the H2-selective mixed matrix membrane (MMM) [52]. The results showed that PSO-ANFIS yielded better predictions in comparison to the other two models yielding R2 = 0.9938 for the testing. In a later work, they applied two intelligent models for the prediction of various gases diffusion through the nanocomposite membranes. They reported that the DE-ANFIS (differential evolution-adaptive neuro-fuzzy inference system) predicted the diffusion of gases more accurately with R2 value of 0.9981 for testing [53]. Rahnama et al. predicted the hydrogen storage capacities in metal hydrides by employing four regression models. They revealed that the boosted decision tree regression model performed better among all the model yielding higher coefficient of determination of 0.83 in comparison to the other three models [54]. In the second study, Rahnama et al. predicted the optimal material groups of metal hydrides using different classification algorithms. The results revealed that the multiclass neural network performed better than the other three algorithms with an accuracy of 80% [55]. 

Among various machine learning algorithms, Support Vector Machine (SVM) can handle datasets with a large number of features and still achieve good classification performance. SVM’s capacity to handle non-linear relationships through kernel methods allows for the capture of complex patterns inherent in hydrogen storage behavior, while its optimization objective mitigates overfitting and enhances generalization performance when validated on independent datasets. Furthermore, SVM’s efficiency in high-dimensional feature spaces enables simultaneous analysis of multiple parameters, reflecting the intricacies of hydrogen storage systems. Using SVM for training and testing, researchers monitor learning relevant to data. They are related to the group of linear classifiers. Meanwhile, the forward destination, which is the classifiers’ unique feature, increases as SVM reduces the experimental classification error. Thus, classifiers with maximum margins were called by SVM. The goal of SVM is to reduce the systemic risk [56]. Therefore, detecting the optimal parameter environment typically requires complete cross-validation. A collection of prototypes is generally referred to as this technique. Model selection is a time-consuming process, which is a practical problem of this process. There are a number of variables involved in the proposed system that can affect the results linked with applying the SVM algorithm. Parameters such as the set of kernel functions, the standard deviation of the Gaussian kernel, the corresponding positions related to the categorized slack variable to hinder the uneven distribution of the categorized outcomes, and the number of training occasions are considered [57]. 

This study proposes a machine learning model that utilizes SVM techniques to predict hydrogen storage classes which are classified on the basis of hydrogen storage capacity values. The input dataset is divided into three classes and each class has its range of hydrogen storage capacity. The hydrogen storage capacity values of less than 1.5 wt.% and from 1.5 wt.% to 3 wt.% are considered as low class and medium class, respectively. The hydrogen storage capacity values beyond 3 wt.% are categorized as high class. For the prediction of hydrogen storage classes, the Hydrogen Storage Prediction using Support Vector Machine (HSP-SVM) was proposed. The proposed HSP-SVM model wa validated using three various techniques such as 5-Fold Cross Validation (5-FCV), Resubstitution Validation (RV), and Holdout Validation (HV). The various statistical parameters were considered to do the comparative analysis of these validation approaches, and the optimal validation approach was identified.

## 2. Simulations and Results

In MATLAB, a proposed HSP-SVM model was implemented on a dataset containing 151,388 samples adopted from the previous study [8]. The used model type for the analysis was SVM, employing the quadratic kernel function. The kernel scale was set to automatic, and box constrain level was kept as one. The multiclass analysis was conducted using one-to-one approach with standardized data as true. All the input features, which are listed in Table 1, were used in the model. For the multiclassification costs, the cost matrix was opted default. The proposed model was evaluated using statistical metrics, including accuracy, Misclassification Rate (MCR), Recall/Sensitivity, True Negative Rate (TNR)/Selectivity, Precision/Positive Predictive Value (PPV), False Positive Rate (FPR), False Negative Rate (FNR), False Discovery Rate (FDR), Negative Predictive Value (NPV), and False Omission Rate (FOR).

Accuracy=HxeTxe+HxgTxgHxeTxe+∑f=1m(Hxf,f≠e)Txf+HxgTxg+∑q=1m(Hxq,q≠g)TxgWhere, e/f/g/q=1, 2, 3, … , m

Missrate=∑q=1m(Hxq,q≠g)Txg∑q=1m(Hxq,q≠g)Txg+HxeTxeWhere, e/g/q=1, 2, 3, … , m

Recall/Sensitivity=HxeTxeHxeTxe+∑q=1m(Hxq,q≠g)TxgWhere, e/g/q=1, 2, 3, … , m

True Negative Rate/Selectivity=HxgTxgHxgTxg +∑f=1m(Hxf,f≠e)Txf Where, f/g=1, 2, 3, … , m

Precision/Positive Predictive value=HxeTxeHxeTxe +∑f=1m(Hxf,f≠e)Txf Where, e/f=1, 2, 3, …, m

$$F_{1}Score=2\times \frac{Precision\times Recall}{Precision+Recall}$$

False Positive Rate=∑f=1m(Hxf,f≠e)TxfHxeTxe +∑f=1m(Hxf,f≠e)Txf Where, e/f=1, 2, 3, … , m

False Discovery Rate=∑f=1m(Hxf,f≠e)TxfHxeTxe +∑f=1m(Hxf,f≠e)Txf Where, e/f=1, 2, 3, … , m

False Omission Rate=∑q=1m(Hxq,q≠g)Txg∑q=1m(Hxq,q≠g)Txg +HxgTxg Where, g/q=1, 2, 3, … , m

Negative Predictive value=HxgTxgHxgTxg +∑q=1m(Hxq,q≠g)Txg Where, g/q=1, 2, 3, … , m

## 3. Materials and Methods

**Figure 1:**
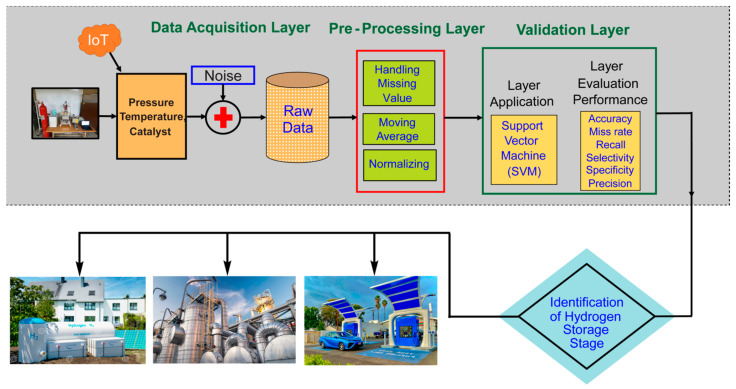
The proposed HSP-SVM model for hydrogen storage prediction.

The proposed Hydrogen Storage Prediction empowered with Support Vector Machine (HSP-SVM) model involves three layers: the data acquisition layer, preprocessing layer, and validation layer, as shown in Figure 1. In the data acquisition layer, devices gather the data of various parameters, but sometimes are missing or have noise due to technical issues or device failures, which is addressed through preprocessing techniques such as handling missing values, moving average methods, and normalization in the data preprocessing layer. After the data preprocessing is completed, the validation layer is activated. This layer is divided into two sub-layers: the application/prediction layer and a performance evaluation layer for calculating various statistical parameters. In the prediction layer, the proposed model uses the SVM algorithm for classification, and three various approaches, such as 5-FCV, RV, and HV, are used for the model validation. The output layer estimates the accuracy, miss rate, recall, precision, and specificity of the proposed HSP-SVM model, as shown in Figure 1.

In this study, the dataset is adopted from the previous study [8] from Figure 4 to Figure 8. The key parameters which directly affect the hydrogen storage capacity of H0-DBT are the temperature and pressure. The hydrogen storage capacity value increases with the increment in temperature and pressure values. Moreover, the catalyst also plays a vital role in accelerating the hydrogen adsorption rates, and optimizing the dosage of the catalyst is imperative. Furthermore, the concentration of H0-DBT may affect the hydrogen storage capacity, and it is necessary to investigate its effect on the attained hydrogen storage capacity. Hence, the selection of key parameters was guided by a comprehensive understanding of the physical and chemical factors influencing hydrogen storage in H0-DBT, aiming to provide insights into the underlying mechanisms governing hydrogen adsorption in H0-DBT. The parameters considered as input and targeted output are listed in Table 1. 

**Table 1:** Input/output parameters for the proposed HSP-SVM model.

S. No.	Input/Output Parameters
Input 1	Temperature
Input 2	Pressure
Input 3	H0-DBT Concentration
Input 4	Catalyst Concentration
Output	Hydrogen Storage Classes (Low, Medium, and High)

The SVM algorithm is a type of machine learning model that is often used for classification tasks involving datasets with many features. It is particularly useful when there are more features than data points. To reduce the amount of memory required, SVM only uses a subset of the training data, called support vectors, in its decision-making process. Various types of kernel functions can be used in SVM, including standard kernels and custom kernels that can be defined by the user. Since we know that the line equation is [58,59]:

a_2_ = ba_1_ + d (1)

where ‘b’ is the slope of a line and ‘d’ is the intersection,

ba_1_ − a_2_ + d = 0

Let $\vec{a}={\left(a_{1},a_{2}\right)}^{t}$ and $\vec{c}=\left(b,-1\right)$, then Equation (1) can be rewritten as

(2) $$\vec{c}\cdot \vec{a}+d=0$$

The equation for a hyperplane in two dimensions is obtained using vectors. The general equation for a hyperplane in any number of dimensions is shown in Equation (2). This equation and the corresponding functions can be used to define the hyperplane in any number of dimensions.

The direction of a vector $\vec{a}={\left(a_{1},a_{2}\right)}^{t}$ is written as c and it is defined as [60]:

(3) $$c=\frac{a_{1}}{||a||}+\frac{a_{2}}{||a||}$$

where

$$\left|\left|a\right|\right|=\sqrt{a_{1+}^{2}a_{2+}^{2}a_{3+}^{2}\ldots \ldots \ldots ..a_{n}^{2}}$$

As we all know,

$$\cos\left(\sigma \right)=\frac{a_{1}}{\left|\left|a\right|\right|}\;\mathrm{and}\;\cos\left(\varphi \right)=\frac{a_{2}}{\left|\left|a\right|\right|}$$

Equation (3) can be written as

$$h=(\cos\left(\sigma \right),\cos\left(\varphi \right))$$

$$\vec{c}\cdot \vec{a}=||c||\;||a||\cos\left(\omega \right)$$

ω=σ−φ

$$\begin{array}{l} \cos\left(\omega \right)=\cos\left(\sigma -\varphi \right)\\ =\cos\left(\sigma \right)\cos\left(\varphi \right)+\sin\left(\sigma \right)\sin\left(\varphi \right)\\ =\frac{c_{1}}{\left|\left|c\right|\right|}\frac{a_{1}}{\left|\left|a\right|\right|}+\frac{c_{2}}{\left|\left|c\right|\right|}\frac{a_{2}}{\left|\left|a\right|\right|}\\ =\frac{c_{1}a_{1}+c_{2}a_{2}}{\left|\left|a\right|||\left|a\right|\right|} \end{array}$$

$$c\cdot a=||c||\;||a||\left[\frac{c_{1}a_{1}+c_{2}a_{2}}{||c||\;||a||}\right]$$

(4) $$\vec{c}\cdot \vec{a}=\sum_{l=1}^{m}c_{l}a_{l}$$

The dot product of two n-dimensional vectors can be computed using the Equation (4).

Let

x=y(c·a+d)

The proposed system measures the performance p on a training dataset, given a dataset D [61,62].

$$x_{l}=y_{l}(c\cdot a+d)$$

The functional margin of a dataset is represented by E, and it shows the degree to which the classes in the dataset are separated from each other. The distance between the hyperplane and the nearest sample from either class is regarded as the functional margin. If the functional margin is large, it reflects that the classes are separated effectively, which, in turn, enhances the performance of the model. The generalization ability of the model is commonly investigated using the functional margin. The model possessing large functional margin will lead to fewer chances of overfitting the training data [63].

$$E=\underset{l=1\ldots ..m}{\min}x_{l}$$

The optimal hyperplane is the hyperplane having the largest functional margin. The prime objective is the identification of the optimal hyperplane, which involves determining the optimal values of the vector (c⃗) and scalar d that define the hyperplane. 

The Lagrangian function shows the following equation [58,59,60]:

$$\delta \left(c,d,b\right)=\frac{1}{2}c\cdot c-\sum_{l=1}^{m}\varphi_{l}[y:\left(c\cdot a+d\right)-1]$$

(5) $$\tau_{j}\delta \left(c,d,b\right)=j-\sum_{l=1}^{m}\varphi_{l}y_{l}a_{l}=0$$

(6) $$\tau_{z}\delta \left(c,d,b\right)=-\sum_{l=1}^{m}\varphi_{l}y_{l}=0$$

From Equations (5) and (6), we obtain:

(7) $$c=\sum_{l=1}^{m}\varphi_{l}y_{l}a_{l}\;\mathrm{and}\;\sum_{l=1}^{m}\varphi_{l}y_{l}=0$$

Substituting the Lagrangian function, δ we obtain:

$$c\left(\varphi ,d\right)=\sum_{l=1}^{m}\varphi_{l}-\frac{1}{2}\sum_{l=1}^{m}\sum_{c=1}^{m}\varphi_{l}\varphi_{l}y_{l}y_{n}a_{l}a_{n}$$

Thus,

(8) $$\underset{\varphi }{\max}\sum_{l=1}^{m}\varphi_{j}-\frac{1}{2}\sum_{l=1}^{n}\sum_{l=1}^{m}\varphi_{l}\varphi_{n}y_{l}y_{n}a_{l}a_{n}$$

Subject to $\varphi_{l}\geq 0,l=1\ldots .m,\sum_{l=1}^{m}\varphi_{l}y_{l}$ = 0.

The Karush–Kuhn–Tucker (KKT) conditions can be extended to the Lagrangian multiplier method when the constraints are unbalanced. The necessary KKT conditions will be expressed as [60,63]:

(9) $$\varphi_{l}\left[y_{l}\left(c_{l}\cdot a^{*}+d\right)-1\right]=0$$

where the optimal point in the dataset is represented by a*, and it is characterized by a positive value of φ. The value of β for all other points in the dataset is approximately zero.

So,

(10) $$y_{l}\left(\left(c_{l}\cdot a^{*}+d\right)-1\right)=0$$

The points in the dataset that are closest to the hyperplane are known as support vectors. The support vectors can be identified using the Equation (10) described above.

$$c-\sum_{l=1}^{m}\varphi_{l}y_{l}a_{l}=0$$

(11) $$c=\sum_{l=1}^{m}\varphi_{l}y_{l}a_{l}$$

To calculate the value of z, we obtain:

(12) $$y_{l}\left(\left(c_{l}\cdot a^{*}+d\right)-1\right)=0$$

Multiplying both sides by e in Equation (12), then it becomes:

$$y_{l}^{2}\left(\left(a_{l}\cdot a^{*}+d\right)-y_{l}\right)=0$$

where $y_{l}^{2}$ = 1.

$$\left(\left(c_{l}\cdot a^{*}+d\right)-y_{l}\right)=0$$

(13) $$d=y_{l}-c_{l}\cdot a^{*}$$

Then:

(14) $$d=\frac{1}{s}\sum_{l=1}^{s}\left(y_{l}-c_{l}\cdot a\right)$$

The number of support vectors, represented by variable A, determines the characteristics of the hyperplane that will be applied to do the predictions. In this manuscript, we examined the application of SVM for multiclass classification. To address this problem, we adopted a strategy of breaking down the multiclass problem into several binary classification problems. Specifically, we employed m × (m − 1)/2 classifiers (where m represents the number of classes) to accomplish the classification task. Therefore, we utilized three classifiers following a one-to-one approach to achieve accurate classification results. And as follows, the hypothesis function is:

(15) $$g\left(c_{i}\right)=\left[\begin{array}{cc} i & ifc_{i}\cdot a+d>{th}_{i}\\ j & else \end{array}\right]$$

In the SVM algorithm used in the proposed HSP-SVM model, points above the hyperplane are classified as class i (in the case of the low-hydrogen storage class: i = 1, in the case of the medium-hydrogen storage class: i = 2, similarity in the case of the high-hydrogen storage class: I = 3); otherwise, point are classified as class j. The goal of the SVM algorithm is to find the optimal hyperplane that can accurately divide the data into the correct classes. The SVM algorithm works by identifying the hyperplane that provides the largest margin, or distance, between the different classes, which helps to improve the accuracy of the model.

## 4. Discussions

### 4.1. 5-Fold Cross Validation

The 5-Fold Cross Validation (5-FCV) approach was evaluated initially. Table 2a–c represents the confusion matrix for the proposed HSP-SVM model with 5-FCV. From Table 2a, it is evident that among storage samples categorized as low class, 37,925 cases were classified accurately as low-class storage. While 105,935 cases were accurately categorized as not belonging to low-class storage, and 7528 cases were erroneously identified as not belonging to low-class storage. It is evident from Table 2b that 57,787 occurrences of medium storage samples were accurately identified as medium-class storage, whereas 9301 instances were wrongly predicted as medium storage. However, 84,300 occurrences of non-medium-class storage were categorized accurately. Table 2c reveals that in the case of high-class storage samples, 46,375 instances were categorized accurately as high-class storage. Whereas 1773 occurrences of non-high-class storage were not identified accurately, and 103,240 samples were accurately classified as not belonging to high-class storage. The results elucidated that the classification accuracy of this approach was 93.90%, with an MCR of 6.10%. The low and medium classes had low accuracies, which resulted in an overall low accuracy for the 5-FCV approach.

### 4.2. Resubstitution Validation

The Resubstitution Validation (RV) approach was used to evaluate the performance of the proposed SVM model for predicting hydrogen storage. Table 2a–c shows the confusion matrix for the proposed SVM model. It is obvious from Table 2a that 37,925 instances were correctly classified as low-class storage, while 7528 samples were wrongly classified as non-class storage. Moreover, 105,935 instances were identified accurately as not belonging to low-class storage. Table 2b shows that 57,787 occurrences of medium-class storage were identified accurately. Whereas 9301 samples were not classified accurately as medium-class storage, and 84,300 entities were identified accurately as non-medium-class storage. In Table 2c, it is revealed that 46,375 instances were identified correctly as high-class storage, while 1773 samples were incorrectly identified as non-high-class storage. Furthermore, 103,240 samples of non-high-class storage were identified accurately. The results revealed that the classification accuracy was 93.90% with an MCR of 6.10%. The overall low accuracy for RV was observed due to high MCR values for the low and medium classes.

### 4.3. Holdout Validation

The third approach was the Holdout Validation (HV) technique that was used to evaluate the proposed HSP-SVM model. The performance of this approach was assessed using a confusion matrix, as shown in Table 3a–c. Table 3a shows that the instances (36,033) in the low storage category were correctly classified, and the instances (87,107) in the non-low storage category were correctly classified, with the exception of 1860 occurrences of non-low-class storage were not categorized accurately. Table 3b shows that in the medium storage category, 48,120 instances were correctly classified, and 3754 instances were wrongly predicted as the medium storage category. However, 73,126 instances were identified accurately as non-medium storage categories. Table 3c shows that in the high storage category, 37,093 instances were correctly classified, and 1894 occurrences of non-high storage category were not identified accurately. Whereas 86,013 instances were predicted accurately as non-high storage categories. The results showed that this approach had a classification accuracy of 97.00% and a misclassification rate of 3.00%. The high accuracy of this approach in classifying low storage capacity was a major contributing factor to its overall performance.

### 4.4. Receiver Operating Characteristic Curve

**Figure 2:**
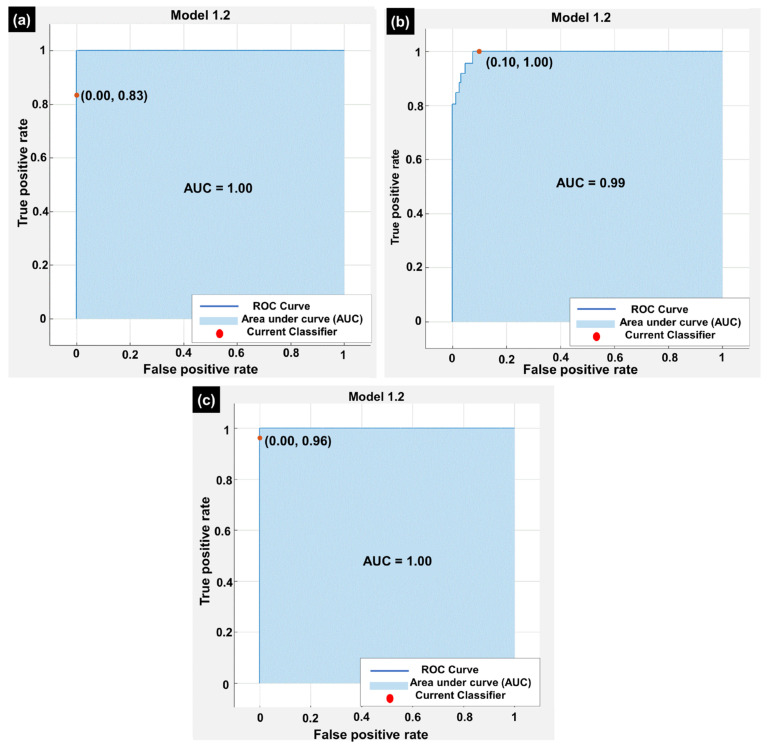
Receiver operating characteristic curves using the 5-FCV, RC, and HV approaches for (**a**) low class, (**b**) medium class, and (**c**) high class.

The receiver operating characteristic (ROC) curves using 5-FCV, RV, and HV for the low class, medium class, and high class are depicted in Figure 2a–c. For the low-class and high-class ROC curves shown in Figure 2a,c, the ROC curves for the classifier in this study showed a True Positive Rate (TPR) of 0.83 and 0.96 and a False Positive Rate (FPR) of 0, respectively. This indicated that the classifier was able to correctly classify 83% and 96% of positive samples and was able to correctly classify all negative samples. The area under the ROC curve (AUC) was calculated to be 1.00, which reveals the perfect performance. It is observed from Figure 2b that the True Positive Rate (TPR) and False Positive Rate (FPR) for the medium class were 1.00 and 0.10, respectively. This elucidates that the classifier performed perfectly for classifying the positive samples whereas 90% of the negative samples were correctly classified. Moreover, the area under the ROC curve (AUC) was 0.99, which elucidates the excellent performance of the classifier. It also reveals that the classifier performed effectively for separating the positive and negative samples. The high TPR and low FPR values emphasize that the classifier achieved a good balance between sensitivity and specificity. Overall, the results revealed that the performance of the classifier was excellent.

### 4.5. Comparative Analysis of the 5-FCV, RV, and HV

**Figure 3:**
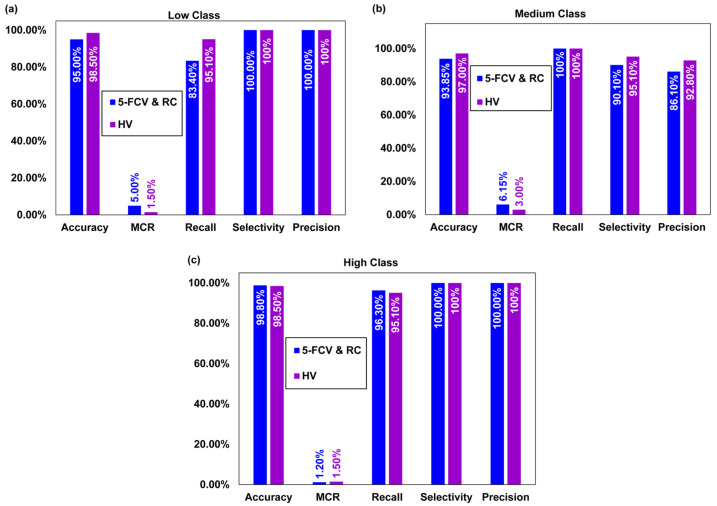
(**a**–**c**) Comparison of statistical parameters for the 5-FCV, RV, and HV approaches.

The 5-FCV, RV, and HV techniques were evaluated using various statistical parameters to assess their performance for all three classes, and results are presented in Table 4. The results showed that the HV model had higher accuracies of 98.5% and 97.0% in comparison to accuracies of 95.0% and 93.8% achieved by the 5-FCV and RV techniques for the low class and the medium class, respectively. Whereas an accuracy of 98.8% obtained by 5-FCV and RV methods and an accuracy of 98.5% obtained by HV were almost similar for the high class. The 5-FCV and RV models yielded a lower misclassification rate (MCR) for the high class (1.20%) compared to the low class (5.00%) and medium class (6.15%). However, the RV approach yielded almost similar MCR for all three classes i.e., 1.50% for the low class and high class and 3.00% for the medium class. The selectivity of all the approaches was similar (100%) for the low and high classes. However, 95.1% was obtained by HV for the medium class, and a lower selectivity of 90.1% was achieved for the medium class from the other two approaches. The recall of all the methods was highest for the medium class (100%), whereas the recall yielded by the HV was higher at 95.1% in comparison to 83.4% yielded by 5-FCV and RV for the low class. All the techniques showed similar precision values of 100% for the low and high classes. However, the HV technique was more precise for the medium class with a precision value of 92.8%. The HV approach yielded higher F1 score values compared to 5-FCV and RV. Specifically, for the low and medium classes, HV yielded F1 scores of 97.5% and 96.2%, respectively, in comparison to the corresponding F1 scores of 90.9% and 92.5% achieved through 5-FCV and RV. However, the F1 score achieved using 5-FCV and RV (98.1%) was slightly higher than of HV (97.5%). The False Positive Rate (FPR) achieved from the HV approach was lower (4.90%) in comparison to the 9.90% yielded by 5-FCV and RV for the medium class. The False Discovery Rate (FDR) was higher for the medium class (13.9%) obtained by 5-FCV and RV, whereas HV yielded an FDR of 4.90% for the medium class. The False Omissions Rate (FOR) was achieved by 5-FCV, and RV was 6.60% for the low class, whereas HV yielded a lower FOR (2.10%) for the low class. The Negative Predictive Value (NPV) was higher (97.90%) for the low class obtained from HV, and it was 93.40% for the low class achieved from 5-FCV and RV approaches. Overall, these results suggested that the HV approach performed best for all three classes compared to the 5-FCV and RV approaches depicted in Figure 3a–c.

**Figure 4:**
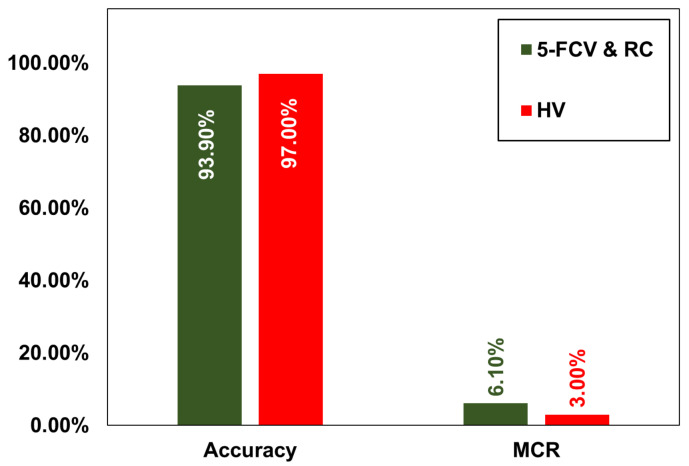
The overall accuracy and MCR of 5-FCV, RC, and HV for hydrogen storage prediction using the proposed HSP-SVM model.

Moreover, the overall accuracy of the HV approach was higher (97.00%) compared to the accuracy (93.90%) of the 5-FCV and RV approaches. Furthermore, MCR was 3.00% for the HV approach, and it was 6.10% for the 5-FCV and RV methods depicted in Figure 4. Hence, the HV approach was found to be optimal to predict the hydrogen storage stages using the proposed HSP-SVM model.

**Figure 5:**
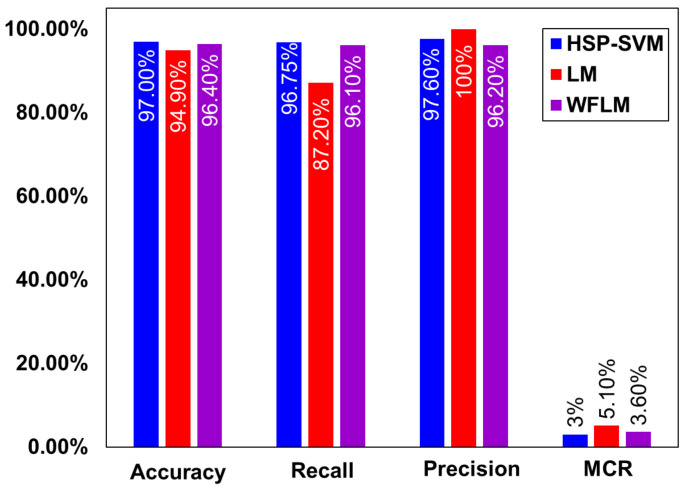
Comparison of the statistical parameters for the proposed HSP-SVM with the LM and WFML models.

As shown in Table 5, our proposed HSP-SVM model exhibited good accuracy compared to previous studies. Moreover, the comparison of the predictive performance of the current model with other classification models, such as Levenberg–Marquardt (LM) [64] and Weighted Federated Machine Learning (WFML) [65], is shown in Figure 5. It is evident from Figure 5 that our current model has performed better in comparison to previously reported classification algorithmic models. The accuracy of HSP-SVM was 97%, whereas it was 94.9% and 96.4% for the LM and WFML models, respectively. Moreover, the recall value for HSP-SVM and WFML was quite close, whereas it was 87.2% for LM.

## 5. Conclusions

Using Dibenzyltoluene (H0-DBT) as a liquid organic hydrogen carrier presents a promising option for hydrogen storage systems. The HSP-SVM model was developed to predict the hydrogen storage classes when storing in H0-DBT, and its performance was validated using various techniques such as 5-FCV, RC, and HV. The HV approach showed a higher accuracy of 97.0%, whereas it was 93.9% for 5-FCV and RC. Moreover, the MCR values for HV, RC, and 5-FCV were 3.00% and 6.10%, respectively. Furthermore, HV approach yielded an accuracy of 98.50% and sensitivity of 95.10%, for the low class in comparison to 95% accuracy and 83.40% sensitivity for the 5-FCV and RC approaches. Similarly, for the medium class, the accuracy and precision of the HV approach were 97% and 92.80%, respectively, whereas the 5-FCV and RC approaches achieved a lower accuracy of 93.85% and sensitivity of 86.10%. Therefore, HV classified the low-class and medium-class data more efficiently than the other two approaches. These results suggested that the HV approach was the optimal approach for the proposed HSP-SVM model to predict hydrogen storage classes in Dibenzyltoluene.

## Figures and Tables

**Table 2:** (a–c). Confusion matrix of the proposed SVM model using 5-Fold Cross Validation and Resubstitution Validation: (a) low class, (b) medium class, (c) high class.

Parameters	Predicted Classes		
	(a) Low Class	(b) Medium Class	(c) High Class
True Positive (TP)	39,725	57,787	46,375
False Negative (FN)	7528	0	1773
False Positive (FP)	0	9301	0
True Negative (TN)	105,935	84,300	103,240

**Table 3:** (a–c). Confusion matrix of the proposed SVM model using Holdout Validation: (a) low class, (b) medium class, (c) high class.

Parameters	Predicted Classes		
	(a) Low Class	(b) Medium Class	(c) High Class
True Positive (TP)	35,033	48,120	37,093
False Negative (FN)	1860	0	1894
False Positive (FP)	0	3754	0
True Negative (TN)	87,107	73,126	86,013

**Table 4:** Comparison of the proposed HSP-SVM model using the 5-FCV, RC, and HV approaches in terms of various statistical parameters.

Evaluation Parameters	5-Fold Cross Validation and Resubstitution Validation			Holdout Validation		
	Low Class	Medium Class	High Class	Low Class	Medium Class	High Class
Accuracy	95.0%	93.8%	98.8%	98.5%	97.0%	98.5%
Miss rate	5.0%	6.15%	1.20%	1.50%	3.00%	1.50%
Selectivity	100%	90.1%	100%	100%	95.1%	100%
Recall/Sensitivity	83.4%	100%	96.3%	95.1%	100%	95.1%
Precision	100%	86.1%	100%	100%	92.8%	100%
F_1_ Score	90.9%	92.5%	98.1%	97.5%	96.2%	97.5%
False positive rate	0	9.90%	0	0	4.90%	0
False discovery rate	0	13.9%	0.00	0	7.20%	0
False omission rate	6.60%	0	1.70%	2.10%	0	2.15%
Negative Predictive Value	93.4%	100%	98.3%	97.9%	100%	97.8%

**Table 5:** Comparison of the current study with previously published studies.

Studies	Year	Storage System	Model	Accuracy
Thornton et al. [66]	2017	Nanoporous materials	Neural Network	88.0%
Rahnama et al. [54]	2019	Metal hydrides	Boosted decision tree regression	83.0%
Rahnama et al. [55]	2019	Metal hydrides	Multiclass neural network	80.0%
Bucior et al. [67]	2019	Metal organic frameworks	Multilinear regression with LASSO [68]	96.0%
Choi et al. [64]	2022	LOHC	Levenberg–Marquardt	94.9%
Ali et al. [65]	2022	LOHC	HSPS-WFML	96.4%
Ali et al.	Current Study	LOHC	HSP-SVM	97.0%

## Data Availability

Data will be available on request.
